# 17β-Estradiol Promotes Trained Immunity in Females Against Sepsis via Regulating Nucleus Translocation of RelB

**DOI:** 10.3389/fimmu.2020.01591

**Published:** 2020-07-22

**Authors:** Zhiheng Sun, Yuchen Pan, Junxing Qu, Yujun Xu, Huan Dou, Yayi Hou

**Affiliations:** ^1^The State Key Laboratory of Pharmaceutical Biotechnology, Division of Immunology, Medical School, Nanjing University, Nanjing, China; ^2^Jiangsu Key Laboratory of Molecular Medicine, Division of Immunology, Medical School, Nanjing University, Nanjing, China

**Keywords:** estradiol, gender difference, macrophages, sepsis, trained immunity

## Abstract

Sepsis is more common among males than females, and the unequal estrogen levels have been suspected to play a vital role in gender differences. Recently, trained immunity is reported to be a novel strategy for the innate immune system to fight infection. However, it has not been clarified whether β-glucan-induced trained immunity causes different responses to early sepsis between male and female mice. In this study, sepsis was induced in mice by intraperitoneal injection of *Escherichia coli* (*E. coli*). The changes of inflammatory cytokines expression, and macrophage polarization in male, female, and ovariectomized C57BL/6 mice in sepsis model were investigated. For *in vitro* studies, different macrophages were treated with LPS. The function of estradiol (E2) on macrophage cell lines was verified and the mechanism of E2 affecting trained immunity was explored. We demonstrated that β-glucan-induced trained immunity was more resistant to sepsis in female than male mice. Macrophage polarization toward the M1 phenotype, which exhibited enhanced trained immunity, was related to the difference in sepsis resistance between female and male mice. Moreover, ovariectomized (OVX) mice manifested serious sepsis consequences with a weaker trained immunity effect than female mice. Female bone marrow-derived macrophages (BMDMs) were also apt to be polarized to the M1 phenotype in response to trained immunity *in vitro*. Furthermore, E2 promoted trained immunity in macrophage cell lines J774 and RAW264.7. E2 was also verified to facilitate trained immunity in primary BMDMs from female and male mice. Mechanistically, we found that E2 inhibited the nuclear translocation of RelB, which is a member of non-canonical pathway of NFκB and contributes to macrophage polarization to change the intensity of trained immunity. This study is the first to indicate the role of E2 in the trained immunity induced by β-glucan to protect against *E. coli*-induced sepsis via the non-canonical NFκB pathway. These results improve our understanding of the molecular mechanisms governing trained immunity in gender differences.

## Highlights

- E_2_ can promote trained immunity to increase sepsis resistance especially in females.
- E_2_ promotes trained immunity by inducing macrophage M1 polarization.
- E_2_ inhibits macrophage M2 polarization by suppressing nucleus translocation of RelB and its downstream gene expression in early stage of sepsis.
- The different E_2_ content *in vivo* may be one of the reasons why men and women have different tolerance to sepsis.

## Introduction

Sepsis is a systemic reaction, which can even be caused by an ordinary infection (1). The infection is most commonly bacterial but also can be fungal, viral, or parasitic. Since many organs are affected, the mortality rate of sepsis is 30–50% (2). With the development of modern medicine, the mortality rates have declined to about 30% (3), but this is still far from acceptable. The biomarker of sepsis is widely discussed by a number of reviews (4–7). Several important signs of sepsis are damage to the kidney, liver, and lung, as well as an increased bacterial load in the kidney and elevated levels of transaminase and lactate in serum (8). Moreover, the common model of sepsis is an immune response to *Escherichia coli* or endotoxin, lipopolysaccharide (LPS), found in the cell wall of gram-negative bacteria. Endotoxin is an excellent example of a pathogen-associated molecular pattern (PAMP). Of note, a number of studies indicate gender dimorphism in terms of response to sepsis (5). The intuitive result is that the incidence of female sepsis is much lower than that of males (9, 10). Furthermore, some studies attributed these differences to the prevailing hormonal milieu of the victim (11–13). Thus, it is necessary to explore the mechanism of estrogen in modulating immunity, which allows females to resist sepsis.

The NFκB (nuclear factor kappa-B) family of transcription factors constitutes five members (RelA or p65, RelB, cRel, NFκB1 or p50, and NFκB2 or p52), all of which play important roles in cell homeostasis, especially in the immune process (14). Apart from the well-known canonical signaling pathway, the non-canonical pathway is also involved in vital immune processes, which could be triggered by signaling from a subset of TNFR members. This pathway mediates the persistent activation of RelB/p52 complex with the ability to modulate a series of gene expression, including macrophage polarization-associated cytokines. To date, a growing number of studies indicated that estrogen is involved in some immune processes. Estrogen can bind to and activate estrogen receptors (ERs), which regulate the expression of downstream genes (15). The interaction between ERs and NFκB is mainly discussed in breast cancer articles (16). A highly significant negative correlation between the expression of NFκB target genes and ER activation was found. Some studies demonstrated the interaction of ER with NFκB; ERs can compete with NFκB for binding to transcriptional coactivators (ie, CREB) or ERs to recruit co-suppressors into NFκB complexes (17). ERs can inhibit *de novo* RelB synthesis in breast cancer tissues and cell lines. In addition, E_2_ inhibits the nucleus translocation of P65, c-Rel, and RelB without affecting P50 in mouse splenocytes (18). These data suggest that estrogen-ER signaling regulates the NFκB pathway at the transcriptional level of its constituents.

The host's immune defense mechanisms can be divided into innate immunity and adaptive immunity. Adaptive immunity, although slower than the innate immune response, has good specificity and produces immunological memory (19). Recently, researchers discovered an ability in innate immunity, similar to immune memory in adaptive immunity, called trained immunity (20). Trained immunity is found in mammals and its basic features have been identified by several researchers. Trained immunity mainly involves a set of cells (myeloid cells, natural killer cells, and innate lymphoid cells) (21). Anti-tuberculosis vaccine Bacillus Calmette-Guérin (BCG) is a well-known immune modulator that induces trained immunity. It was reported that estrogen did not influence the induction of trained immunity by BCG, and did not induce training or tolerance in monocytes themselves, indicating that the estrogen is unlikely to explain the sex-differential effects after BCG vaccination (22). In fact, β-glucan is a major cell wall component of *C. albicans* and can induce trained immunity in monocytes. The initiation of trained immunity is associated with enhanced signaling of the Akt (protein kinase B)-mTOR (mammalian target of rapamycin)-HIF-1α (hypoxia-inducible factor-1α) pathway (23), modifications in metabolic pathways (conversion to glycolysis), and epigenetic rewriting (24, 25). However, the effect of estrogen on β-glucan-induced trained immunity to resist sepsis has not been clarified.

## Materials and Methods

### Reagents and Antibodies

β-glucan (G-59303) and β-Estradiol (50-28-2) were purchased from XiEnSi biotechnology company (Tianjin, China). M-CSF (#CB34), IFN-γ (#C746), and TNFα (#CF09) were purchased from Novoprotein (Shanghai, China). E_2_ and LPS was purchased from Sigma (St. Louis, MO, USA). Antibody against p-Akt (Ser473, #4060), Akt (#4691), p-4EBP1 (#2855), 4EBP1 (Thr37/46, #9644T), p-S6 (Ser235/236, #4858), S6 (#2217), PCNA (#13110), P65 (#8242), Estrogen receptor α (13258s), GAPDH (#5174), and β-Actin (#3700) were purchased from Cell Signaling (Boston, MO, USA). RelB (sc-166416) was purchased from Santa Cruz Biotechnology (Dallas, TX, USA). F4/80-APC, F4/80-FITC, CD116-PE, CD206-APC, CD206-FITC, iNOS-PE were all from Biolegend (San Diego, CA, USA).

### Cell Culture

Raw264.7, J774, and HEK293T cells were obtained from the Type Culture Collection of the Chinese Academy of Sciences, Shanghai, China. Cells were cultured in phenol red-free DMEM with 10% FBS, 1% (100 U/mL penicillin and 100 ug/mL streptomycin) at 37°C in an atmosphere of 95% air and 5% CO_2_. Cells were seeded onto different types of plates for further experiments when the cell density reached ~80%. The cells were used within a maximum of five passages.

The *in vitro* trained immunity model was established with RAW264.7 and J774. Cells were challenged with 5 μg/mL β-glucan for 24 h. The cells were then washed and rested in culture medium for 5 days. Next, cells were treated with 100 ng/mL LPS for 6 h, and then the cytokines were measured in mRNA level.

1 × 10^5^ BMDMs were seeded in 96-well plates (200 μL final volume; Corning) and stimulated with 100 μg/mL β-glucan for 24 h. Then cells were washed and rested for 3 days in culture medium. On day 4, BMDMs were washed again and treated with 25 ng/mL IFN-γ for 24 h. On day 5, a final wash was performed, and cells were primed with 1 μg/mL LPS. The supernatants were harvested for ELISA assay after 24 h of LPS stimulation. Other experiments were all based on the same model but with different numbers of BMDMs. To assess mRNA expression and cell viability, 6 × 10^5^ BMDMs were plated in non-treated 24-well plates (1,200 mL final volume; Corning) and followed the training scheme described above. For western blotting (WB) assays 3 × 10^6^ BMDMs were plated in six-well plates (3 mL final volume; Corning).

### Mice

Male and female C57BL/B mice were purchased from the Model Animal Research Center of Nanjing University. Mice used in the model of trained immunity and sepsis were 10 weeks old. All animal procedures were performed in accordance with guidelines of the US NIH with Specific Pathogen Free conditions. Soy-free standard rodent chow and water were provided *ad libitum*. Female mice were anesthetized with 4% chloral hydrate and then underwent ovariectomy (OVX) at 6 weeks of age. When they were 10 weeks old, the same model of trained immunity and sepsis was established.

### PBMC Isolation

Peripheral blood mononuclear cells (PBMCs) were separated from mouse plasma by Ficoll centrifugation using lymphocyte separation medium from MP Biomedicals (Solon, USA) according to the standard procedures.

### BMDM Isolation and Culture

Mice were sacrificed via cervical dislocation and sterilized by soaking in 75% ethanol. Dissected the legs and bone marrow was extracted from tibia and femur bones by using a 25-gauge needle and a 1 mL syringe filled with PBS following removal of surrounding muscle. Blow the bone marrow gently and spread it through a 70 μm cell strainer. The cell suspension was centrifuged at 300 g for 5 min at room temperature. Cells were cultured in phenol red-free DMEM with 10% ultra-low endotoxin FBS and M-CSF (20 ng/mL). After changing the fresh medium on the third and fifth day, BMDM was induced.

### *E. coli* Strain Preparations

The *E. coli* 15597 strains were purchased from ATCC, collected, and identified by the Medical Laboratory Center of Zhongda Hospital in Nanjing Jiangsu, China, and stored at −80°C. Bacterial strains were prepared in LB medium.

### Nuclear Protein Extraction

A nuclear protein extraction kit was purchased from Biyuntian (Wuhan, China) and used according to the manufacturer's instructions.

### siRNA Transfection

siRNA was transfected according to the product instructions (Ruibo company, China). The concentration of siRNA used in the study was 50 nM. The ERα siRNA target sequence is TGCACATTGAAGATGCTGA. The target sequence of non-coding (NC) siRNA was a random sequence with no biological effects.

### Cell Viability Assay

To assess the effect of E_2_ on cell viability, a CCK-8 assay was used according to the manufacturer's instructions (Bioss company, China). RAW264.7/J774 were seeded onto 96-well plates at a concentration of ~5 × 10^4^ cells/well. Different concentrations of E_2_ were used to treat cells for different length of time as described in the article.

### Quantitative PCR

Total RNA was extracted from cells using TRIzol reagent, and reverse transcriptions were performed in a 20 μL mixture with 1 μg of total RNA according to the manufacturer's instructions (Vazyme company, China). The oligonucleotide primers used for PCR amplification are listed in Table 1. PCR amplification consisted of 30 cycles of denaturation at 95°C for 2 min, annealing at 60°C for 45 s, and extension at 72°C for 2 min. All reactions were run in triplicate. The gene expression levels were normalized to ß-actin.

**Table 1:** Primer sequences.

**Gene**	**Sense (5^**′**^-3^**′**^)**	**Anti-sense (5^**′**^-3^**′**^)**
TNFα	CAGCAAGGGACAGCAGAGG	AGTATGTGAGAGGAAGAGAACC
IL-1B	GCAACTGTTCCTGAACTCAACT	ATCTTTTGGGGTCCGTCAACT
IL-6	TAGTCCTTCCTACCCCAATTTCC	TTGGTCCTTAGCCACTCCTTC
IL-10	GCTCTTACTGACTGGCATGAG	CGCAGCTCTAGGAGCATGTG
IL-4	GGTCTCAACCCCCAGCTAGT	GCCGATGATCTCTCTCAAGTGAT
ERα	CCTCCCGCCTTCTACAGGT	CACACGGCACAGTAGCGAG
BACT	GGCTGTATTCCCCTCCATCG	CCAGTTGGTAACAATGCCATGT

### Western Blotting

The protein samples were obtained from lysis buffer treated cells. Cell lysates were put on ice for 15 min and then centrifuged at 12,000 × g for 10 min. Subsequently, 30 μg of protein per lane was separated on 10% polyacrylamide gels and transferred onto polyvinylidene difluoride membranes (Millipore, Billerica, MA, USA). Membranes were blocked with 5% bovine serum albumin (BSA) in Tris-buffered saline containing 0.1% Tween 20, and then the membranes were incubated with specific antibodies. The values were normalized to the β-actin/GAPDH intensity levels.

### *In vivo* Models

Mice were trained with two intraperitoneal (i.p.) injections of 1 mg β-glucan particles on days −7 and −4. Sterile PBS was used as a control. On day 0, mice were challenged with 1.77 × 10^7^ *E. coli*. The lung, kidney, and serum were harvested 24 h after *E. coli* treatment.

### H&E Staining

The fresh lung tissues were fixed in 4% paraformaldehyde (PFA). Then, the samples were gradually dehydrated and embedded in paraffin. After that, the samples were cut into 3 μm sections and stained with hematoxylin and eosin for further light microscopy observation. Scores were evaluated by a pathologist based on the lung tissue integrity, alveolar integrity, and mononuclear infiltration (0 = none; 1 = mild; 2 = moderate; 3 = severe).

### Immunofluorescence

Cultured cells were seeded on glass coverslips in six-well plates. After three PBS washes, the samples were fixed for 15 min at room temperature with 4% paraformaldehyde. Fixed cells were rinsed with PBS and then incubated for 10 min at 4°C with 0.2% Triton X-100 and 0.2% BSA in PBS. Following permeabilization, non-specific binding in the cells was blocked by 5% BSA in PBS for 1 h at room temperature. Cell samples were incubated with anti-ERα, anti-RelB, and anti-p65 primary antibodies at a 1:200 dilution for 2 h at room temperature. Samples were further incubated with Alexa Fluor 488-conjugated and Alexa 647-conjugated secondary antibody at a 1:400 dilution for 1.5 h in the dark. After washed with PBS, the nuclei were stained by DAPI. Slides were visualized using a Nikon Eclipse Ti-U fluorescence microscope equipped with a digital camera (DS-Ri1, Nikon).

### ELISA

The protein concentration of IL-6, lactate, TNFα, and estrogen in cell supernatant or mouse serum were detected using the corresponding mouse enzyme-linked immunosorbent assay (ELISA) kit according to the manufacturer's instructions (Biolegend, China).

### Kidney Burden

The kidney *E. coli* burden at indicated time points was measured by plating organ homogenates obtained mechanically over 70 μm cell strainers (BD Biosciences) following slicing the tissue, in serial dilutions on LB agar plates; colony-forming units (CFUs) were counted after growth at 37°C for 24 h, and data are shown as CFUs in total kidney.

### Flow Cytometry Analysis

PBMCs or BMDMs were filtered through a 70 μm cell strainer and then washed with complete RPMI medium to generate single-cell suspensions. An Fc-receptor blocker (CD16/32, eBioscience) was used to reduce non-specific antibody binding. Antibodies used in these experiments included F4/80-APC, F4/80-FITC, iNOS-PE, CD206-APC, and CD11b-PE. Stained samples were detected by a FACS Calibur flow cytometer (BD Bioscience) and data were analyzed using FlowJo software (TreeStar, Ashland, OR).

### Statistical Analysis

The statistical analysis was performed using Prism (Prism 5 for Windows, GraphPad Software Inc., USA). Unless specified, statistical significance for comparison between two sample groups with a normal distribution (Shapiro-Wilk test for normality) was determined using two-tailed paired or unpaired Student's *t*-test. Differences were considered significant at *p* < 0.05 as indicated. Except when specified, only significant differences are shown. As indicated in figure legends, either a representative experiment or a pool is shown, and the number of repetitions of each experiment and number of experimental units (either cultures or mice) is indicated. The results are presented as the means ± standard error (SEM).

## Results

### β-Glucan-Induced Trained Immunity Makes Female More Resistant to Sepsis Than Male Mice

**Figure 1:**
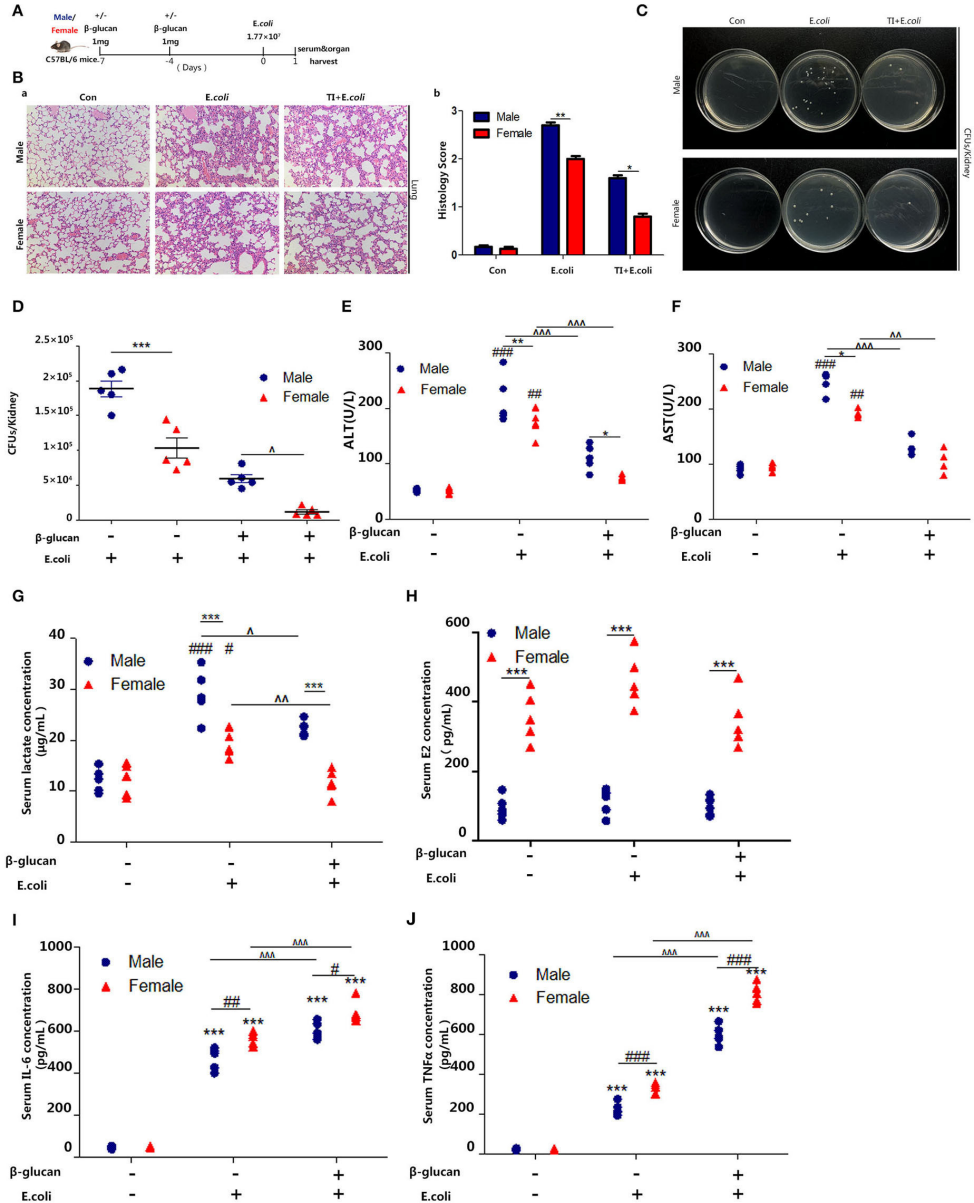
β-glucan induced trained immunity is more resistant to sepsis in female than male mice. **(A)** *In vivo* model of trained immunity (TI) by two intraperitoneal (i.p.) β-glucan injections and secondary i.p. *E.coli* challenge (*n* = 5/group). **(B)** Histological analysis of the lung was visualized by H&E staining. The lung was microscopically analyzed and histologically scored by a pathologist. **(C)** Kidney homogenates after dilution were plated on LB agar plates at 37°C for 24 h to count CFUs. **(D)** Kidney *E.coli* burden at indicated time points was shown in the mouse model as **(A)**. **(E,F)** Levels of serum ALT and AST were detected in female and male mice treated with *E. coli* or TI + *E. coli* groups. **(G)** Serum lactate concentration was detected by ELISA in female and male mice treated with *E. coli* or TI + *E. coli* groups. **(H)** Serum concentration of estradiol (E_2_) was analyzed by ELISA in *E. coli* and TI + *E. coli* groups. **(I,J)** Serum IL-6 and TNFα were measured by ELISA to characterize the extent of the immune response. Each panel is representative of at least three independent biological replicates. In **(D–J)**, single dots correspond to individual mice. ^#^*p* < 0.05, ^##^*p* < 0.01, and ^###^*p* < 0.001, paired Student's *t*-test comparing *E. coli* group, TI + *E. coli* group and control group in same gender. *p < 0.05, **p < 0.01, and ***p < 0.001, paired Student's *t*-test comparing in the same experimental group. ∧ *p* < 0.05, ∧∧*p* < 0.01, and ∧∧∧*p* < 0.001, paired Student's *t*-test comparing *E. coli* group and TI + *E. coli* group in same gender.

Several studies have shown that women have better survival and tolerance to sepsis than men (26, 27). Thus, we further verified this phenomenon by establishing a sepsis model in mice (Figure 1A). The difference in lung injury between male and female mice in sepsis was determined by H&E analysis of lung sections (Figure 1Ba). The histochemical scores (Figure 1Bb) indicated that male mice had more severe lung damage than female mice; trained immunity prevented lung injury in mice and the protective effect of trained immunity was better for females than males. Notably, we observed a decreased renal *E. coli* burden in females than males. In addition, in trained immunity mice, females had less renal *E. coli* burden than males (Figures 1C,D). In addition, liver damage markers, aspartate aminotransferase (AST), and alanine aminotransferase (ALT) were increased in males than females; and trained immunity markedly decreased AST and ALT concentration, with a greater decrease in females than in males (Figures 1E,F). We found that in the sepsis model, the serum lactate concentration of the male mice was higher than that of the female mice. In the trained immunity group, serum lactate was decreased, with the level in the females lower than males (Figure 1G). As expected, male serum E_2_ content is much lower than female mice (Figure 1H). Our results showed that females expressed higher IL-6 and TNFα than males in sepsis, and trained immunity exacerbated this trend (Figures 1I,J).

### Macrophage Polarization Toward M1 Phenotype Is Associated With Different Resistance to Sepsis Between Female and Male Mice

**Figure 2:**
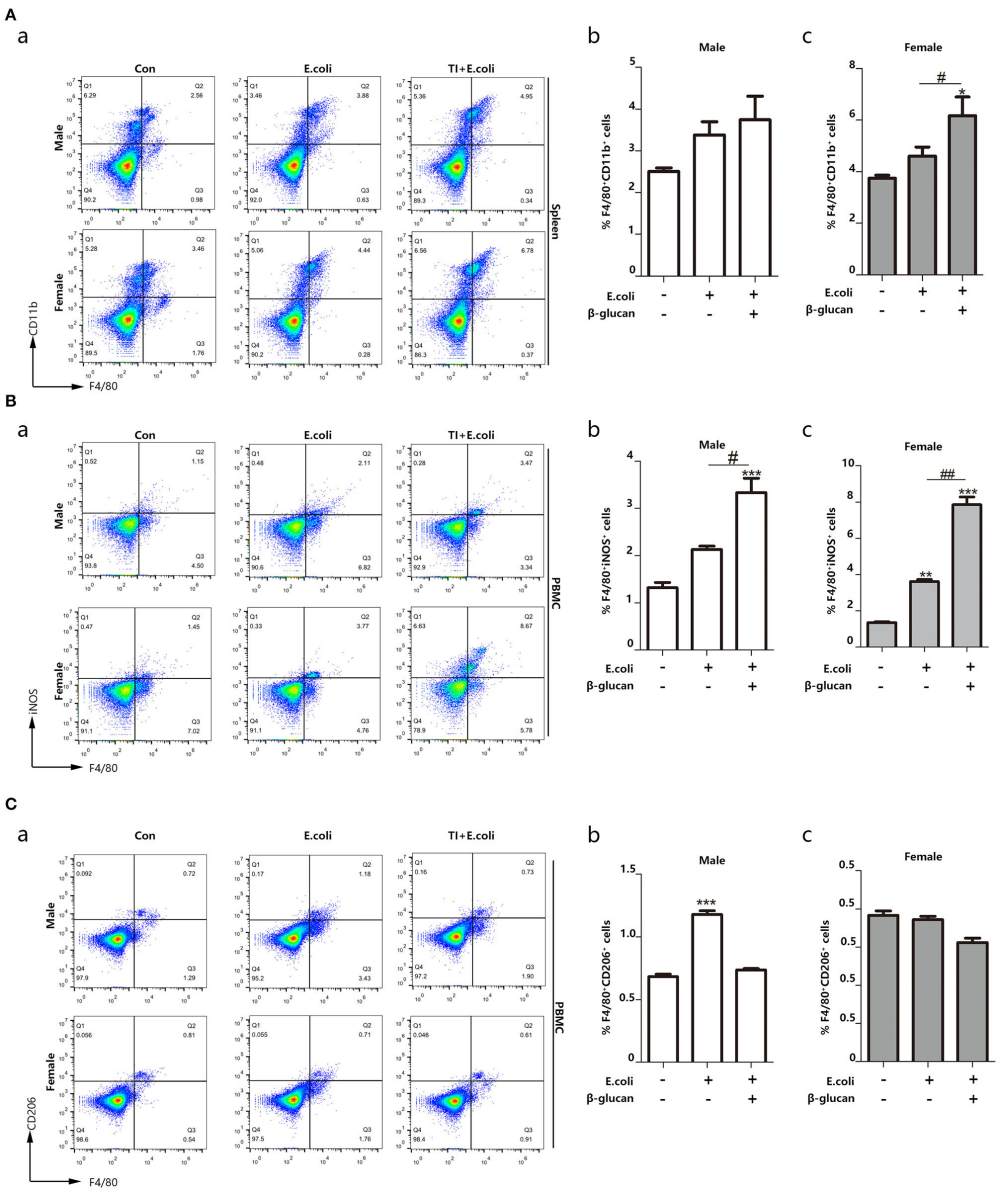
Macrophage polarization toward M1 phenotype is related to different resistance to sepsis between female and male mice. The enhanced trained immunity exhibited the increased M1 phenotype macrophage. **(A)** The percentage of F4/80^+^CD11b^+^ marked macrophage was significantly increased in the spleen from both *E. coli* and TI + *E. coli* groups (*n* = 3/group). **(B)** The percentage of F4/80^+^iNOS^+^ M1 macrophage was significantly increased in PBMCs from both *E. coli* and TI + *E. coli* groups (*n* = 3/group). **(C)** The percentage of F4/80^+^CD206^+^ M2 macrophage in PBMCs from both *E. coli* and TI + *E. coli* groups (*n* = 3/group). Data with error bars are presented as the mean± standard error (SEM). Each panel is a representative experiment of at least three independent biological replicates. *p < 0.05, **p < 0.01, and ***p < 0.001, paired Student's *t*-test comparing *E. coli* group, TI + *E. coli* group and control group in same gender. ^#^*p* < 0.05, ^##^*p* < 0.01, paired Student's *t*-test comparing *E. coli* group and TI + *E. coli* group.

Macrophages are the most significant cells in the body in regard to trained immunity (28). The promoted trained immunity is related to the increased M1 phenotype macrophage. In order to understand the different response to sepsis between the different genders, we further investigated the polarization of macrophages during the process. Thus, we focused on macrophage content and polarization. By detecting the percentage of macrophages in the spleen, we found that macrophages in female mice are more abundant than male mice, and trained immunity significantly increased the content of female macrophages (Figure 2A). Sepsis promoted the polarization of macrophages to M1 and trained immunity aggravated this trend. Notably, females showed more changes than males (Figure 2B). On the other hand, females inhibited the polarization of M2 macrophages (Figure 2C). Trained immunity simulated M1-type polarization of macrophages, and this effect was more pronounced for females.

### OVX Mice Shows Serious Septic Consequences With a Weaker Trained Immunity Effect Than Female Mice

**Figure 3:**
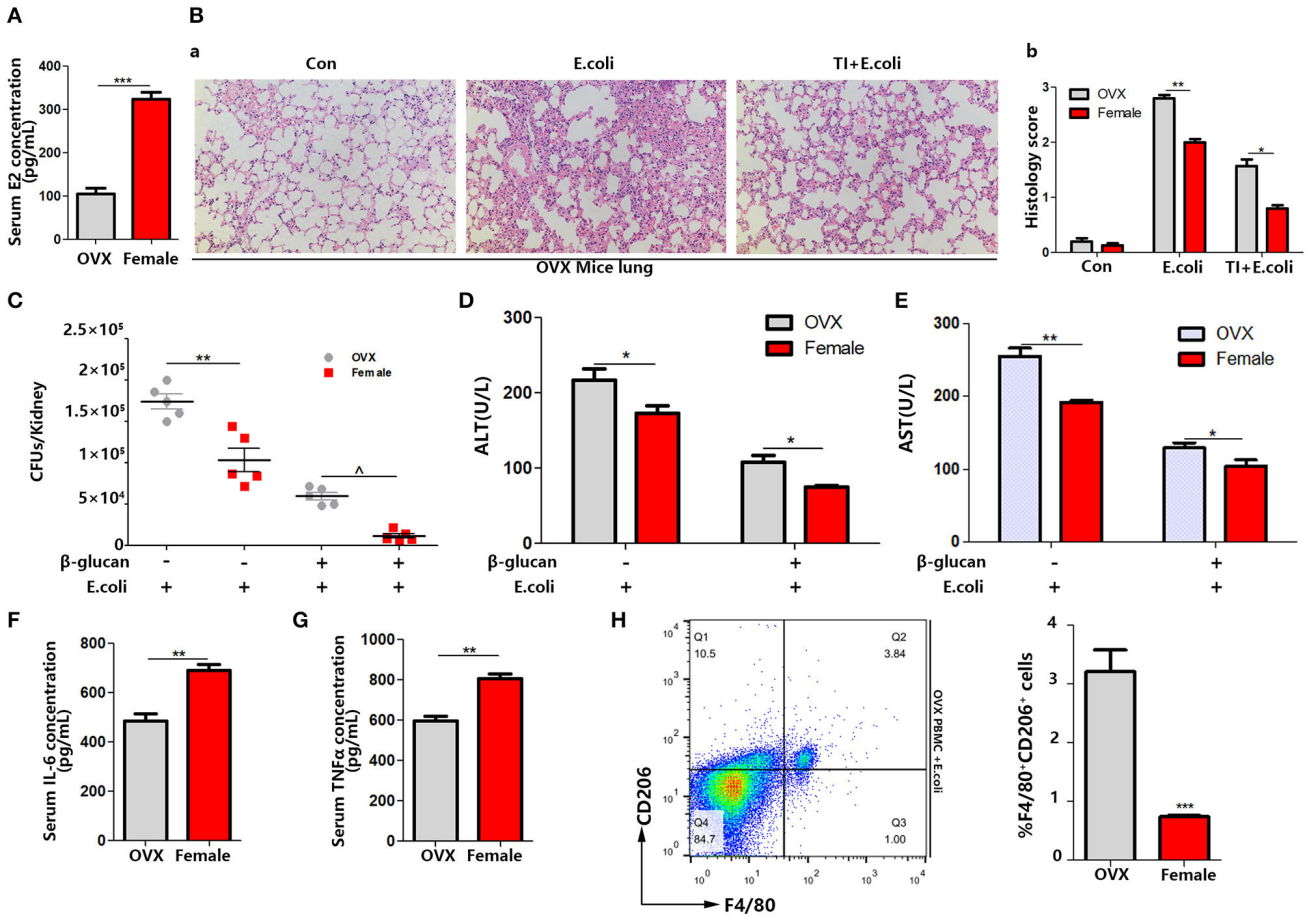
OVX mice manifest serious sepsis consequences with a weaker trained immunity effect than female mice. **(A)** Serum E_2_ concentration was significantly decreased in OVX mice. **(B)** The lung was visualized by H&E staining, microscopically analyzed, and histologically scored by a pathologist. **(C)** Kidney *E. coli* burden in OVX mice compared with female mice at indicated time points following model in Figure 1A. **(D,E)** Level of serum ALT and AST were detected in OVX and female mice treated with *E. coli* or TI + *E. coli* groups. **(F,G)** Serum IL-6 and TNFα were measured by ELISA to characterize the different extent of the trained immunity response between OVX and female mice. **(H)** PBMCs from OVX mice trended toward M2 polarization in *E. coli* group compared to female mice (*n* = 5/group). *p < 0.05, **p < 0.01, and ***p < 0.001 as determined by Student's *t*-test.

By analyzing the experiments above, we found that the difference between male and female responses to sepsis was due to the difference in macrophage polarization *in vivo*, and the difference in estrogen content in the different genders was very significant. To verify that differences in the estrogen level play a vital role, OVX mice were made, since the ovary is the main organ to produce E_2_. The same sepsis model was established with OVX mice (Figure 1A). Firstly, the serum E_2_ concentration was significantly decreased (Figure 3A). The degree of lung injury was much higher than that of female mice as well as TI + *E. coli* group (Figure 3B). The kidney *E. coli* burden was also evaluated. The results showed that OVX mice also had more severe kidney injury (Figure 3C) as well as lung injury, as measured by serum ALT (Figure 3D) and AST (Figure 3E) concentrations, in comparison to female mice. With a lower serum IL-6 and TNFα concentration in OVX mice in the TI + *E. coli* group, the data suggested that OVX mice have a lower trained immunity response than female mice (Figures 3F,G). Finally, similar to male mice, OVX mouse PBMCs began polarizing to M2-type at 24 h after i.p. *E. coli* (Figure 3H). Taken together, these results demonstrated that E_2_ does facilitate trained immunity in the body to resist sepsis, and simultaneously inhibits macrophage polarization to M2.

### Female BMDMs in Trained Immunity Are More Inclined to M1-Type Polarization *in vitro*

**Figure 4:**
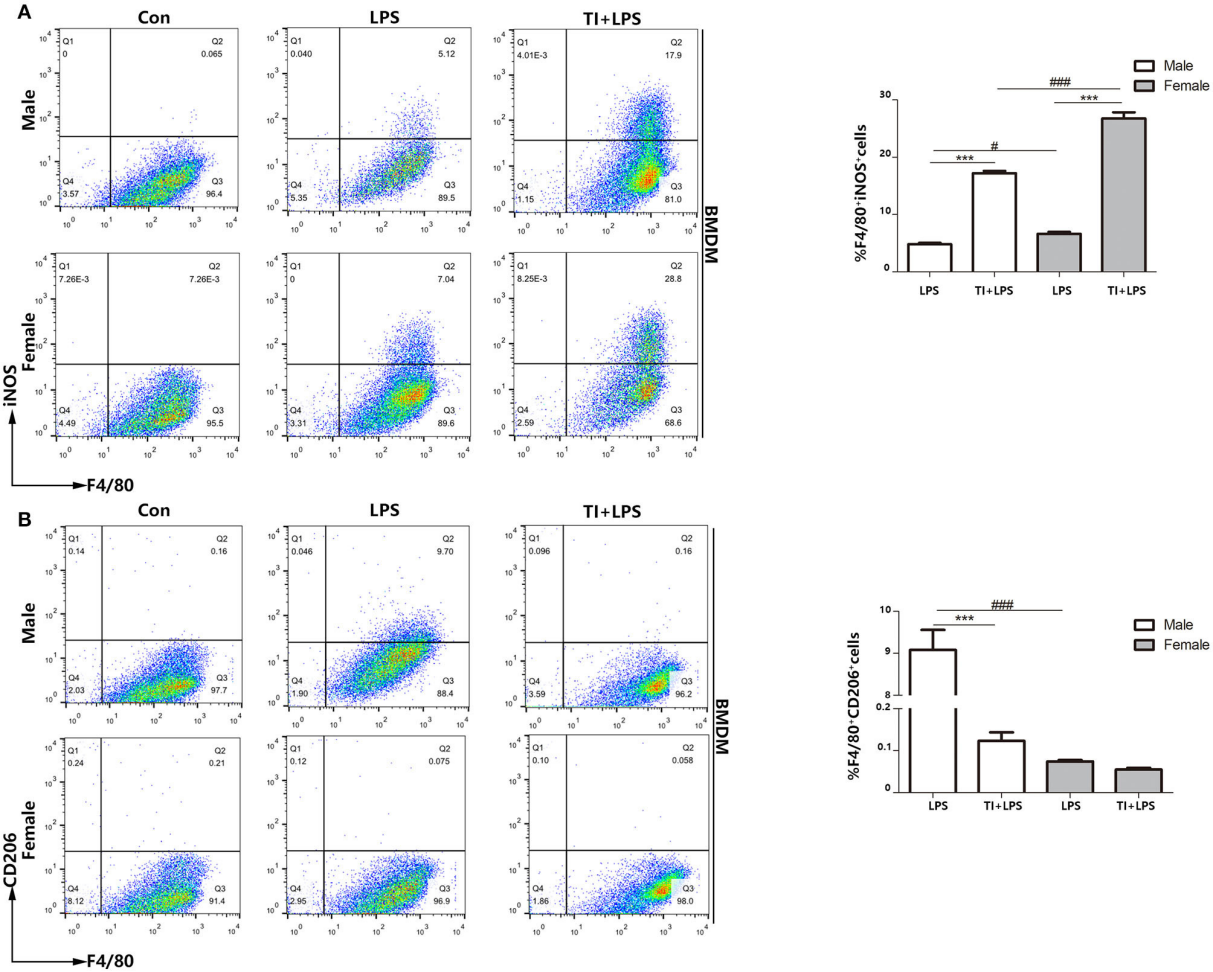
Female BMDMs are apt to be polarized to M1 phenotype in responses to trained immunity *in vitro*. **(A)** Female BMDMs M1 polarization was stronger than male BMDMs treated with LPS or TI + LPS groups. **(B)** Very few female BMDMs to polarized to M2 in LPS group, while male BMDMs showed the polarization of M2 phenotype. TI suppressed BMDMs to M2 polarization from both male and female mice (*n* ≥ 3/group). ***p < 0.001, paired Student's *t*-test comparing LPS group and TI + LPS group in the same gender. ^#^*p* < 0.05, ^###^*p* < 0.001, paired Student's *t*-test comparing same group between different genders.

Since the macrophages are mostly derived from bone marrow cells, and also the cytokines up-regulated in trained immunity are pro-inflammatory cytokines. Although the upregulation of inflammatory cytokines after trained immunity of macrophages is determined, no research has studied the polarization of macrophages during this process. Therefore, exploring the polarization of BMDM as an important primary macrophage in trained immunity is very significant and necessary to understand the nature of trained immunity. To determine whether BMDMs are similar to macrophage polarization in PBMCs, we tested the polarization of microphages from male or female mice when challenged with LPS or trained immunity plus LPS. We considered F4/80^+^iNOS^+^ BMDMs as M1-type BMDMs and F4/80^+^CD206^+^ BMDMs as M2-type BMDMs. The results demonstrated that female BMDMs are more polarized to M1 in both the LPS group (7.04–5.12%) and TI + LPS group (28.8–17.9%) (Figure 4A). Also, male BMDMs began to transform to M2 in 24 h, which did not occur in female BMDMs. Trained immunity also prevented both male and female BMDMs from M2 polarization as well as PBMCs (Figure 4B).

### E_2_ Facilitates Trained Immunity in Macrophage Cell Lines J774 and RAW264.7

**Figure 5:**
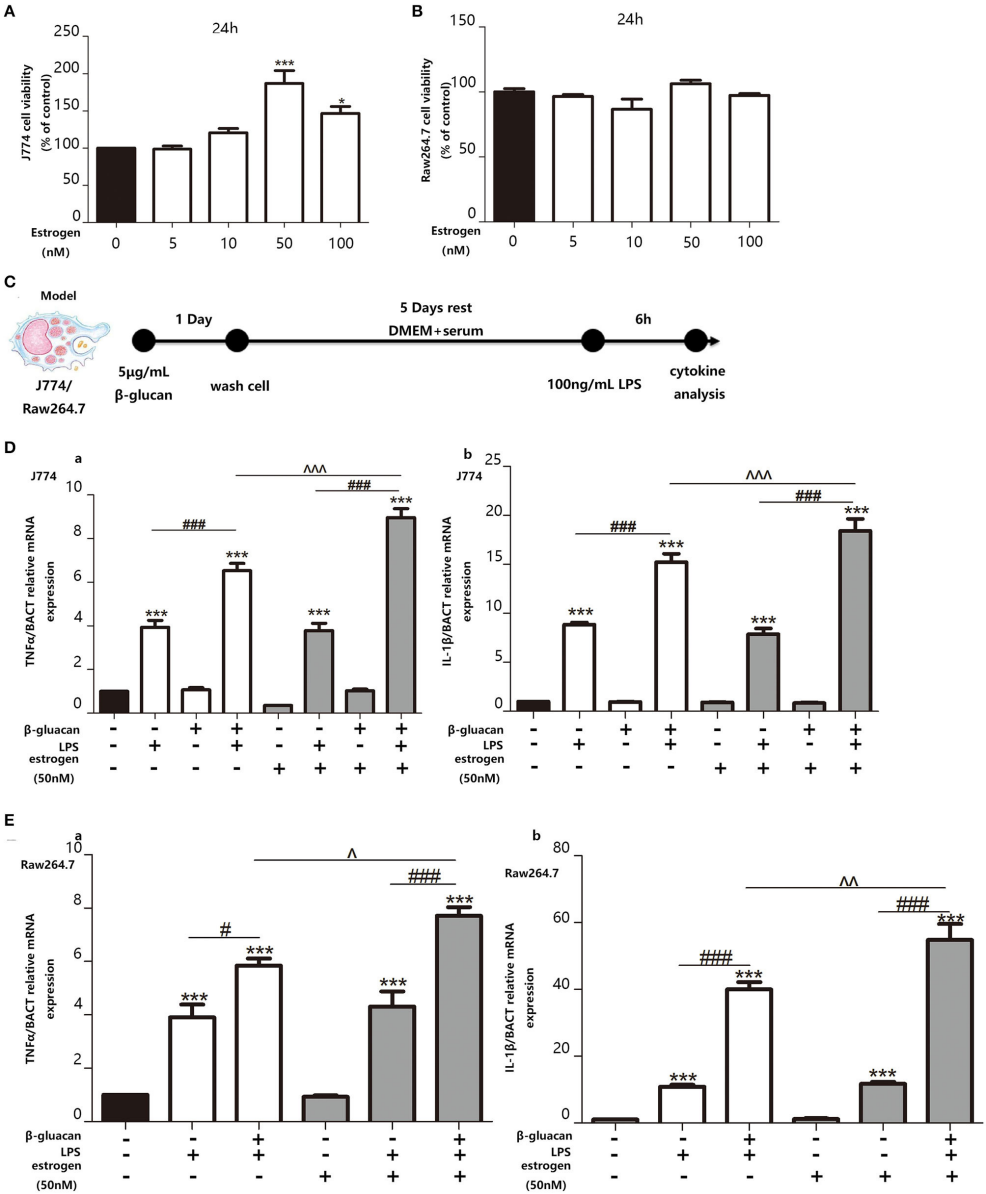
E_2_ promotes trained immunity in macrophage cell lines J774 and RAW264.7. **(A,B)** The effect of E_2_ on both J774 and RAW264.7 cell viability for 24 h was tested with CCK-8 assay kit. **(C)** *In vitro* trained immunity model for J774 and RAW264.7 cell line. **(D)** The mRNA levels of TNFα and IL-1β in J774 were detected by qPCR to determine the trained immunity effect with or without E_2_. **(E)** The mRNA levels of TNFα and IL-1β in RAW264.7 were detected by qPCR to determine the trained immunity effect with or without E_2_ (*n* ≥ 3/group). ^#^*p* < 0.05, ^###^*p* < 0.001, paired Student's *t*-test comparing β-glucan + LPS group and LPS group. *p < 0.05, ***p < 0.001, paired Student's *t*-test comparing with control group. ∧*p* < 0.05, ∧∧*p* < 0.01, and ∧∧∧*p* < 0.001, paired Student's *t*-test comparing between β-glucan + LPS groups with or without E_2_.

In order to examine the effect of estradiol on trained immunity, we decided to pre-treat the macrophage cell lines with estradiol before trained immunity model. Since the concentration of estradiol that can stimulate the macrophage cell lines RAW264.7 and J774 is unknown, we made a concentration gradient of estradiol on the activity of the macrophage cell lines. The results showed that the response of the macrophage cell lines to the stimulation of estradiol is not particularly sensitive, and the viability of J774 begins to increase significantly under the stimulation of 50 nM estradiol. Thus, the concentration of E_2_ used in cell experiments was verified as 50 nM (Figures 5A,B). The *in vitro* trained immunity model was established with RAW264.7 and J774 (Figure 5C) cell lines derived from male and female mice, respectively. Using TNFα and IL-1β as the marker of inflammation, our data suggested that E_2_ can induce stronger trained immunity both in RAW264.7 and J774 (Figures 5D,E).

### E_2_ Facilitates Trained Immunity in Primary BMDMs From Female and Male Mice

**Figure 6:**
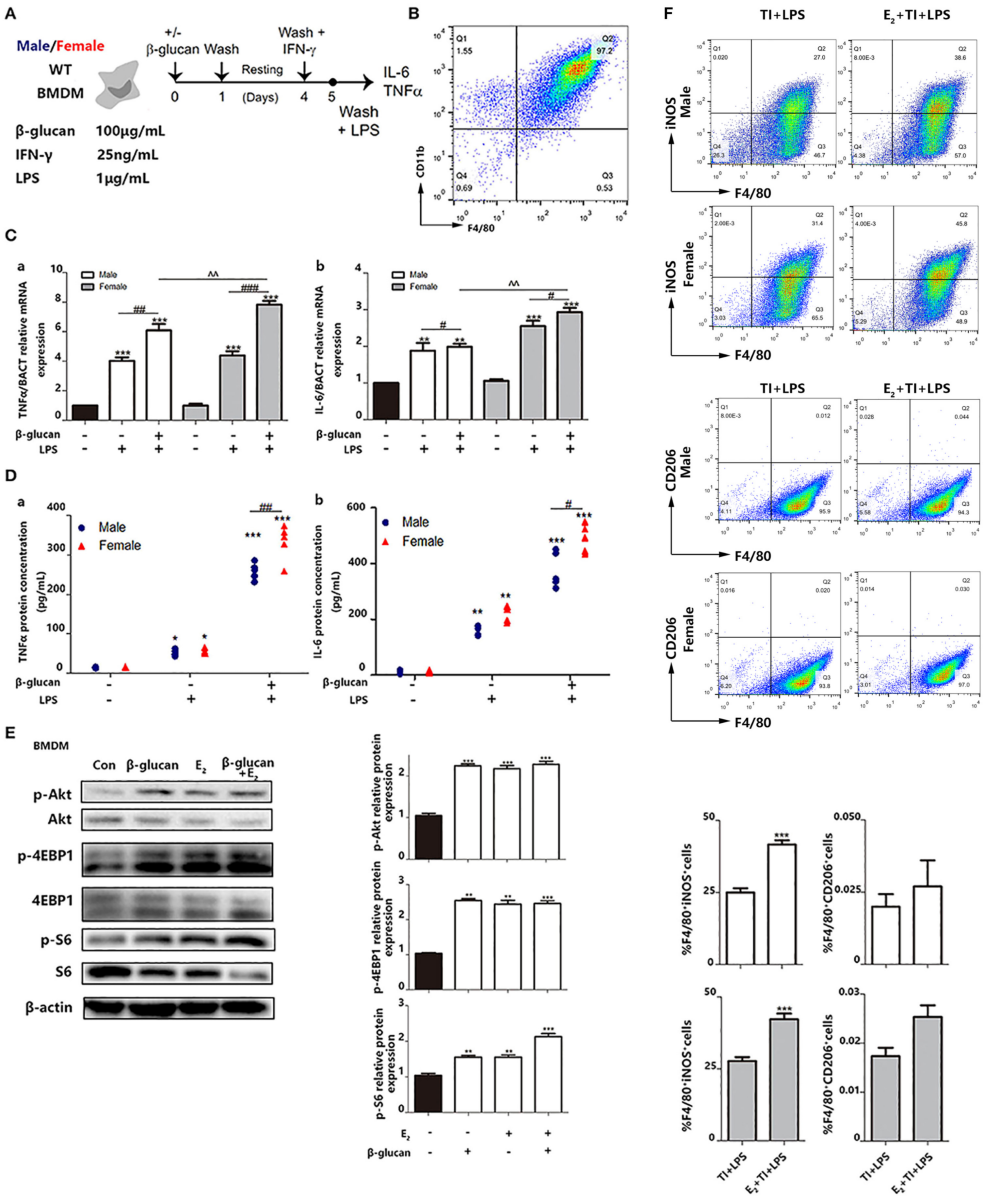
E_2_ is verified to facilitate trained immunity in primary BMDMs from female and male mice. **(A)** *In vitro* trained immunity model for BMDMs. **(B)** Flow cytometry was used for testing the purity of BMDMs induced by *in vitro* culture. **(C)** The mRNA levels of TNFα and IL-6 in male/female BMDMs were detected by qPCR to determine the different intensity of trained immunity between genders. **(D)** The protein concentrations of TNFα and IL-6 from the supernatant from male/female BMDM cultures were detected by ELISA to determine the different intensity of trained immunity between genders. **(E)** E_2_ activated hallmarks of trained immunity, such as Akt, 4EBP1, and S6 by western blot. **(F)** E_2_ promoted M1 polarization in TI + LPS group from male and female mice. Meanwhile, E_2_ maintained the M2 polarization to inhibit the effect of TI (*n* ≥ 3/group). ^#^*p* < 0.05, ^##^*p* < 0.01, and ^###^*p* < 0.001, paired Student's *t*-test comparing β-glucan + LPS group and LPS group. *p < 0.05, **p < 0.01, and ***p < 0.001, paired Student's *t*-test comparing with control group. ∧∧*p* < 0.01, paired Student's *t*-test comparing between β-glucan + LPS groups with or without E_2_.

We also made a trained immunity model of BMDMs (Figure 6A). M-CSF was used to induce BMDM with a purity of over 95% from both male and female mice (Figure 6B). Then, the mRNA levels (Figure 6C) and protein levels (Figure 6D) of TNFα and IL-6 were measured. The results suggested that female BMDMs can have a more intensive response to LPS as well as a trained immunity response than male BMDMs. Phosphorylation of Akt and mTOR targets (S6 and 4EBP1) are considered to be key hallmarks of trained immunity (23). To clarify the effect of E_2_ on trained immunity, these hallmarks are checked by western blot, which indicated that E_2_ as well as β-glucan induced trained immunity in BMDMS (Figure 6E). In addition, the data showed that E_2_ further boosted the polarization of M1-type macrophage in trained immunity. Consistently, this effect of E_2_ is more apparent in female BMDMs, and E_2_ inhibited M2 polarization in trained immunity (Figure 6F).

### E_2_ Inhibits Nuclear Translocation of RelB to Modulate Macrophage Polarization, Which Regulates the Intensity of Trained Immunity

**Figure 7:**
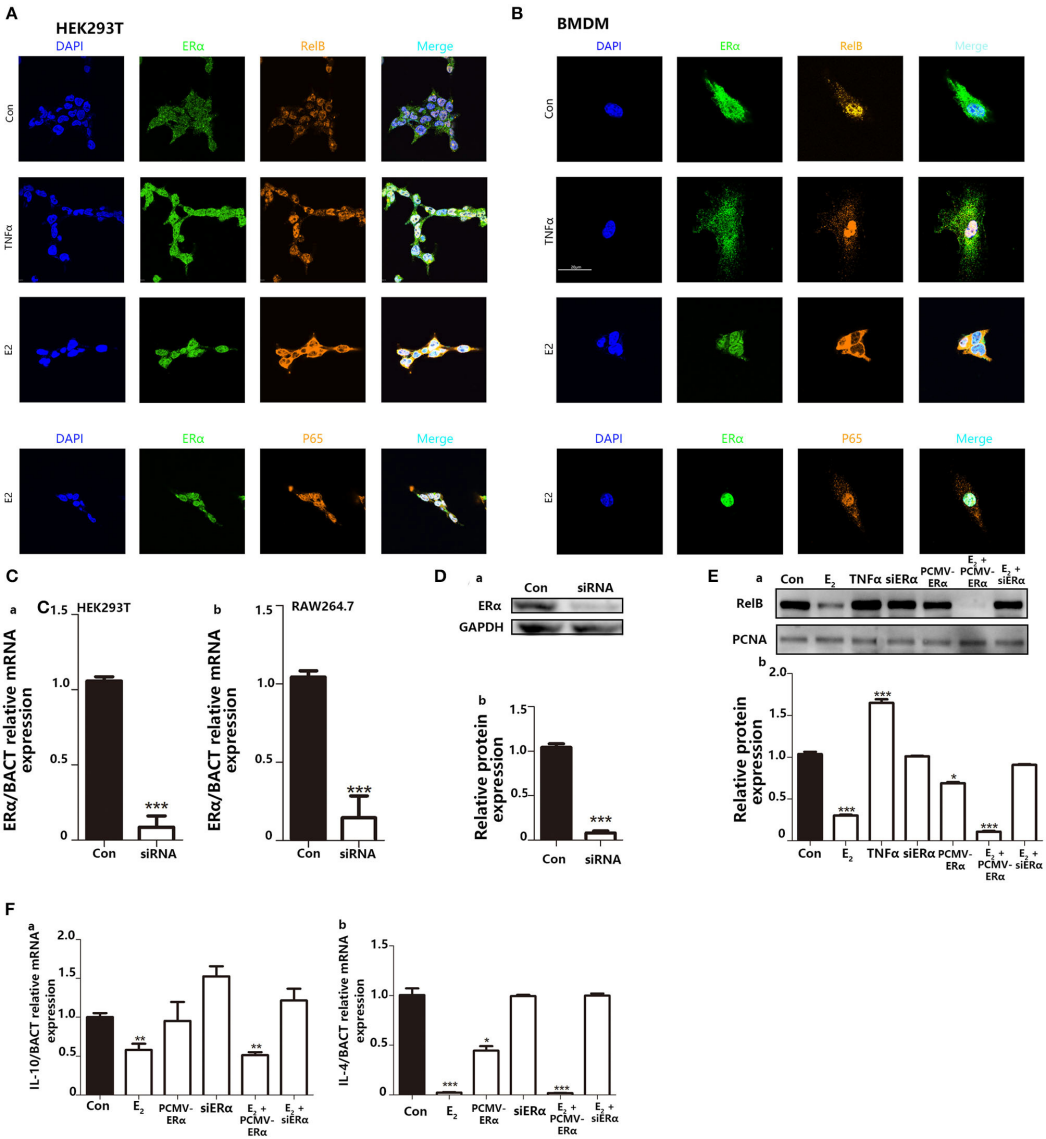
Inhibition of E_2_ on nuclear translocation of RelB contributes to macrophage polarization to change the intensity of trained immunity. **(A)** Immunofluorescence indicated that E_2_ treatment inhibited nucleus translocation of RelB but not P65 in the HEK293T cell line. **(B)** Immunofluorescence indicated that E_2_ treatment inhibited nuclear translocation of RelB but not P65 in BMDMs. **(C)** The knockdown function of ERα siRNA was verified by testing the ERα mRNA level in HEK293T and RAW264.7. **(D)** The knockdown function of ERα siRNA was verified by testing ERα protein expression level in RAW264.7. **(E)** Nuclear protein was extracted to determine the effect of E_2_ on the content of RelB in nuclear in RAW264.7. **(F)** The effect of E_2_ on the downstream gene of RelB, IL-4, and IL-10, in RAW264.7 was confirmed by PCR (*n* ≥ 3/group). *p < 0.05, **p < 0.01, and ***p < 0.001, paired Student's *t*-test comparing with control group.

NFκB signaling pathway is involved in the regulation of many cellular physiological processes. Previous articles have reported that RelB^−/−^ mice, or inhibiting the nucleus translocation of RelB, led to a higher basal inflammatory level (29, 30). Other reviews declared that E_2_ could regulate nuclear translocation of NFκB through ERs. Since it has been widely studied that TNFα can combine with TNFR on the cell membrane surface to promote NFκB translocate into the nucleus. Also, because TNFα as a representative inflammatory cytokine is secreted by macrophages and will stimulate back to macrophage. We use TNFα as a positive control to study NFκB entry into the nucleus. We use immunofluorescence to explore the intracellular localization of NFκB protein. In immunofluorescence, the results indicated that when TNFα is used to stimulate cells, it does not affect the colocalization of ERα and the nucleus but increases the colocalization area of P65 and RelB with the nucleus. On the contrary, E_2_ treatment appeared to induce nuclear positioning of ERα and kept RelB distribution in the cytoplasm in both HEK293T and BMDMs (Figures 7A,B). However, stimulation of E_2_ did not affect the distribution of P65 in cells. Next, we used the RNA interference system to further verify the impact of E_2_ on the nuclear translocation of RelB. The silencing performance of interfering small RNA sequence was evaluated both at the mRNA level (Figure 7C) and protein level (Figure 7D). By extracting nuclear protein, we found that estrogen could reduce the content of RelB protein in the nucleus; by silencing the expression of ERα, the content of RelB protein in the nucleus was also restored (Figure 7E). As markers of M2 macrophage polarization, IL-4 and IL-10 are also the downstream genes regulated by RelB (30, 31). We found that E_2_ inhibited the mRNA expression level of IL-10 and IL-4 by regulating nuclear translocation of RelB (Figure 7F). In conclusion, these results indicated that E_2_ could block RelB translocation to the nucleus; to further inhibit M2-associated gene expression, E_2_ suppressed BMDMs M2 polarization.

## Discussion

During the development of sepsis, many systemic organ abnormalities occur including damage to the lung, liver and kidney; the normal operation of the body's neurosensory system may even be affected (32). Since LPS is a component in the cell wall of gram-negative bacteria, it is wildly used as an ideal stimulus to establish sepsis models. When TLR4 on the macrophage cell membrane recognizes LPS, a series of signaling pathways will be initiated to stimulate macrophages to produce pro-inflammatory cytokines, which can induce potent systemic inflammatory responses *in vivo*. Therefore, TNFα, IL-1β, and IL-6 are currently considered to be markers of the early phase of sepsis (33). The development of sepsis begins with systemic inflammatory response syndrome (SIRS) (34), which is considered as the early phase of sepsis. Elevated expression of pro-inflammatory cytokines could help the body return to a normal state in early sepsis (35). After SIRS, the body enters the CARS (compensatory anti-inflammatory response syndrome) stage. In the CARS stage, the immune system will undergo the decline-regulation (36). The hyper-inflammatory response of SIRS will normally be restored by the subsequent CARS stage. The patients can return to normal state after the CARS stage (37). However, if the CARS phase fails, it can cause death from sepsis. As one of the important ways to protect the body from foreign microbes and antigens, the role of trained immunity is very significant. This study focused on the effects of E_2_ on early sepsis by promoting trained immunity. In the development of early sepsis, estrogen induced a stronger trained immunity response of macrophages to remove antigens and microorganisms from the body. Simultaneously, estrogen promoted the polarization of macrophages to M1 *in vivo* and inhibited the polarization of M2, which is considered anti-inflammatory in the early phase of sepsis. In summary, estrogen can maintain the higher pro-inflammatory state of macrophages. This also illustrates the reason why females are more tolerant to sepsis than males in one aspect. However, further research is needed to understand the role of estrogen in the later stages of sepsis.

Here, we demonstrated that β-glucan-induced trained immunity was more resistant to sepsis in female than male mice. OVX mice manifested serious sepsis consequences with a weaker trained immunity effect than female mice. Macrophage polarization toward M1 phenotype is thought to exhibit the enhanced trained immunity. E_2_ promoted trained immunity *in vitro* and *in vivo*. Inhibition of E2 on the nucleus translocation of RelB contributed to macrophage polarization to change the intensity of trained immunity. Our results indicated that E_2_ is involved in the trained immunity in gender differences.

Since trained immunity was discovered in 2014, although there are many articles about trained immunity, there is no article to find the fundamental way of trained immunity activation but only about the changes of immune cell characterization after trained immunity activation. Such as epigenetic changes, upregulation of glycolysis, autophagy level, Akt phosphorylation, and so on. In this study, we found that E_2_ promotes trained immunity by suppressing RelB entry into nucleus, while inhibiting macrophage polarization to M2 in inflammation (A way reverse the inflammatory response). It is possible that E_2_ can change the epigenetics of macrophages through estrogen receptors to affect the trained immunity. This hypothesis needs further researches.

Trained immunity is a way of immunization that occurs in the innate immune cells through the first stimulation to fight the later secondary infections. By obtaining trained immunity, the body can fight various infections from bacteria, fungi and even viruses timely. It is worth mentioning that in countries and regions with underdeveloped scientific research capabilities, it may be an economical and quick means of protection against Covid-19 to enhance people's trained immunity ability. Sepsis is a clinically common infectious disease with obvious gender differences. In this article, sepsis model is used as an infection model to evaluate the role of estradiol in enhancing trained immunity. The mechanism and effects of estradiol in promoting trained immunity also need subsequent study in other types of infectious diseases.

The purpose of this paper was to explore the reasons why different genders have different tolerance effects on sepsis. An article reported that estradiol and DHT (dihydrotestosterone) did not influence BCG-induced trained immunity, and the article only focused on the inflammatory cytokines of monocytes induced by BCG *in vitro* (22). Apart from this, almost no articles study the effects of E_2_ on trained immunity and its biological significance. Our research attempted to explain its effects on trained immunity and understand how it causes women to be more tolerant of sepsis than men. However, in terms of gender differences, many factors, such as androgen concentration, genetic differences, metabolic differences, and physiological differences may all be the result of different tolerances for sepsis between genders (12, 38, 39). Gender differences are a very complex and exquisite scientific issue (40), and all of the factors that contribute to gender differences are worth exploring for subsequent experiments.

## Conclusion

In summary, we demonstrated that as one of the key factors for gender differences, E_2_ plays a vital role in the stronger tolerance of females to sepsis than males. One the one hand, E_2_ can better facilitate trained immunity by increasing expression of inflammatory cytokines and inducing macrophage M1-type polarization; on the other hand, E_2_ can inhibit or delay the macrophage M2-type polarization by regulating nucleus translocation of RelB and its down-stream M2-associated genes. Thus, this may provide a new idea for the clinical treatment of sepsis. Drugs with a structure similar to estrogen may be developed to treat sepsis. More importantly, the treatment of some diseases may able to find better solutions by addressing the different characteristics of different genders.

## Data Availability Statement

The datasets generated for this study are available on request to the corresponding author.

## Ethics Statement

The animal study was reviewed and approved by Model Animal Research Center of Nanjing University.

## Author Contributions

All authors reviewed the manuscript, contributed to data analysis, drafting and revising the article, gave final approval of the version to be published. ZS conceived and designed the project. ZS, YP, JQ, and YX participated in the animal experiments. ZS and JQ wrote the manuscript. YH and YX revised it.

## Conflict of Interest

The authors declare that the research was conducted in the absence of any commercial or financial relationships that could be construed as a potential conflict of interest.

## Abbreviations

E_2_: 17-β-estradiol
TI: trained immunity
LPS: lipopolysaccharide
PBS: phosphate buffer saline
AST: aspartate aminotransferase
ALT: alanine aminotransferase
IL-6: interleukin-6
TNFα: tumor necrosis factor α
IL-10: interleukin-10
IL-4: interleukin-4
M-CSF: macrophage colony-stimulating factor.
