# Corrigendum: 17β-Estradiol promotes trained immunity in female against sepsis via regulating nucleus translocation of RelB

**DOI:** 10.3389/fimmu.2025.1629629

**Published:** 2025-06-13

**Authors:** Zhiheng Sun, Yuchen Pan, Junxing Qu, Yujun Xu, Huan Dou, Yayi Hou

**Affiliations:** ^1^ The State Key Laboratory of Pharmaceutical Biotechnology, Division of Immunology, Medical School, Nanjing University, Nanjing, China; ^2^ Jiangsu Key Laboratory of Molecular Medicine, Division of Immunology, Medical School, Nanjing University, Nanjing, China

**Keywords:** estradiol, gender difference, macrophages, sepsis, trained immunity

In the original article, there was a mistake in **Figure 6F** as published. *The incorrect flow-cytometry results pictures were used in E2+TI+LPS group due to the inconsistent use of gating strategy and the misuse of the same picture.* The corrected **Figure 6F** appears below.

**Figure 6:**
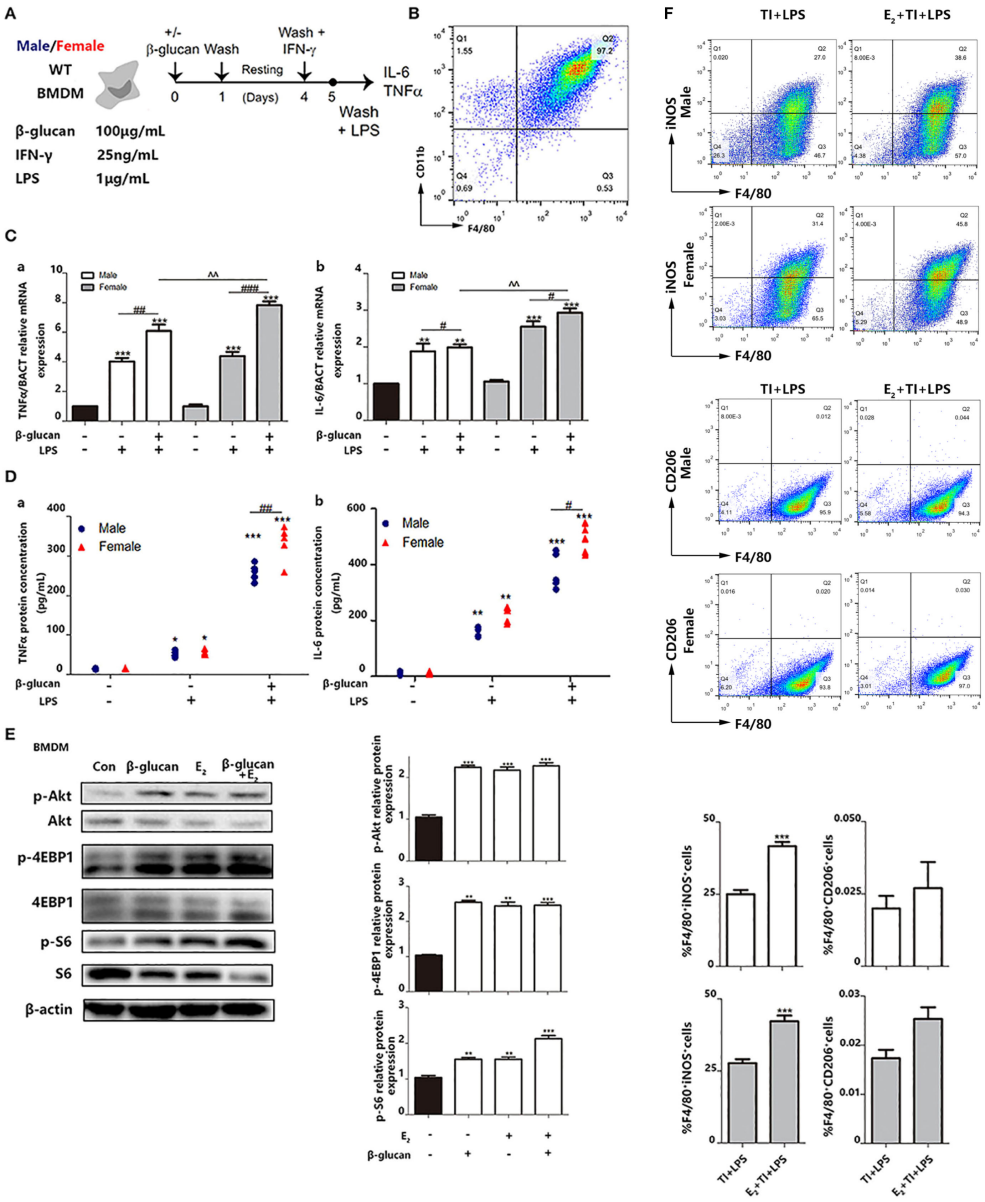
E_2_ is verified to facilitate trained immunity in primary BMDMs from female and male mice. **(A)** In vitro trained immunity model for BMDMs. **(B)** Flow cytometry was used for testing the purity of BMDMs induced by in vitro culture. **(C)** The mRNA levels of TNFα and IL-6 in male/female BMDMs were detected by qPCR to determine the different intensity of trained immunity between genders. **(D)** The protein concentrations of TNFα and IL-6 from the supernatant from male/female BMDM cultures were detected by ELISA to determine the different intensity of trained immunity between genders. **(E)** E_2_ activated hallmarks of trained immunity, such as Akt, 4EBP1, and S6 by western blot. **(F)** E2 promoted M1 polarization in TI + LPS group from male and female mice. Meanwhile, E_2_ maintained the M2 polarization to inhibit the effect of TI (n ≥ 3/group). ^#^p < 0.05, ^##^p < 0.01, and ^###^p < 0.001, paired Student’s t-test comparing β-glucan + LPS group and LPS group. *p < 0.05, **p < 0.01, and ***p < 0.001, paired Student's t-test comparing with control group. ^^p < 0.01, paired Student’s t-test comparing between β-glucan + LPS groups with or without E_2_.

The authors apologize for this error and state that this does not change the scientific conclusions of the article in any way. The original article has been updated.

